# Supplementary material for: Causal involvement of dorsomedial prefrontal cortex in learning the predictability of observable actions
Source: Nat Commun. 2024 Sep 27;15:8305. doi: 10.1038/s41467-024-52559-0 (PMC11436984; doi:10.1038/s41467-024-52559-0)
Supplement: Supplementary file 1 — Supplementary Information [file 41467_2024_52559_MOESM1_ESM.pdf]

Supplementary Information

Supplementary Results

Response times (RTs)

Linear mixed models showed that participants took longer to choose with the bad demonstrator than with the superb demonstrator, b = 0.157, t_{40}= 3.061, p< 0.005, Bayes $factor_{10}$ = 1.06 (Figure S1). Our planned pairwise comparisons for stimulation site revealed no significant results for choice in all conditions.

Supplementary Figures

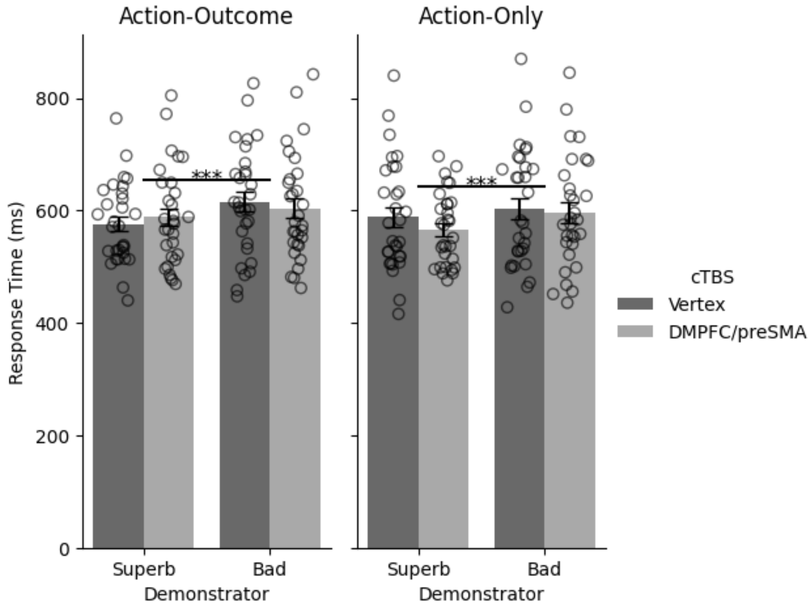

Figure S1. *Choice for self* response times. Compared to vertex stimulation, there was little evidence to suggest that DMPFC/preSMA downregulation affected how long it took participants to choose one of the two options. However, responses were faster when participants observed the superb demonstrator rather than the bad demonstrator, $b = 0.157$, $t_{40} = 3.061$, $p = 0.003$. *** indicates $p < 0.005$. Error bars indicate the standard error (SE).

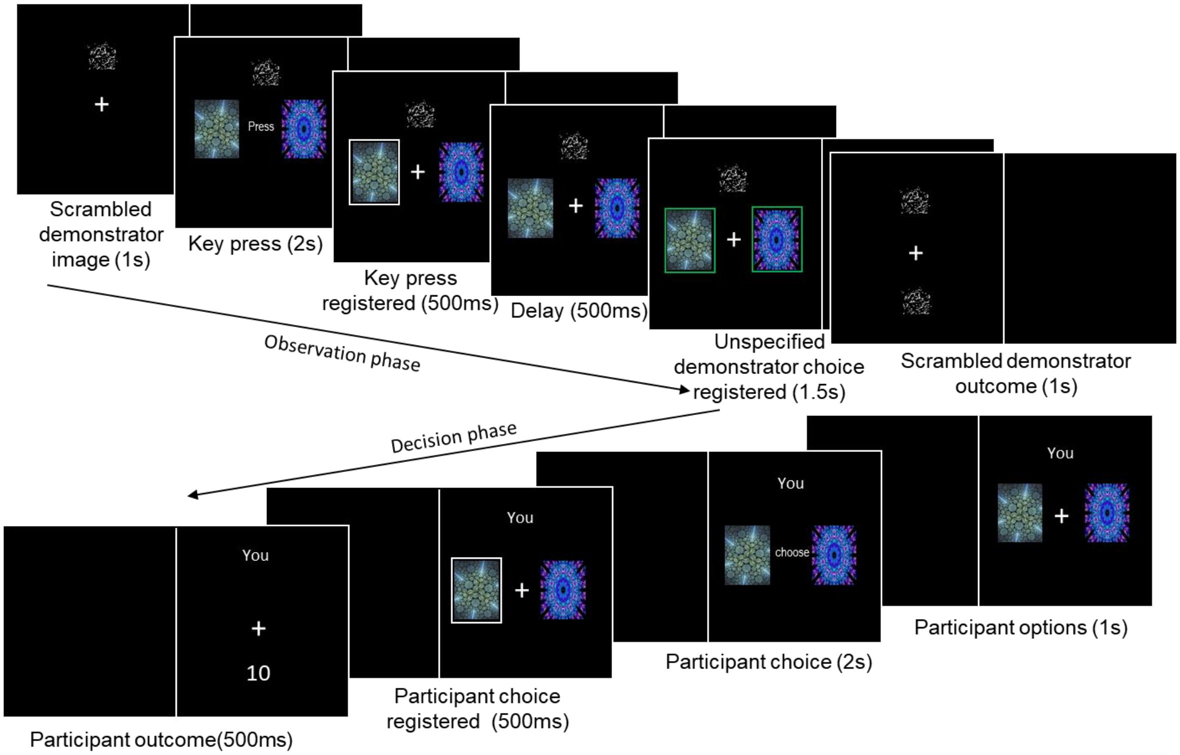

Figure S2. Trial structure of the individual learning task. The photo of the demonstrator was replaced by a scrambled picture in the observation phase. Instead of predicting which option the demonstrator would choose, participants were asked to press one of the two keys. The choice of the demonstrator remained unspecified by framing both images. The demonstrator outcome was scrambled. In the decision phase, the same pair of fractal images was displayed. The participant chose one of the images and finally viewed their outcome. Thus, participants were forced to learn the probabilities of each option from their own actions and outcomes.

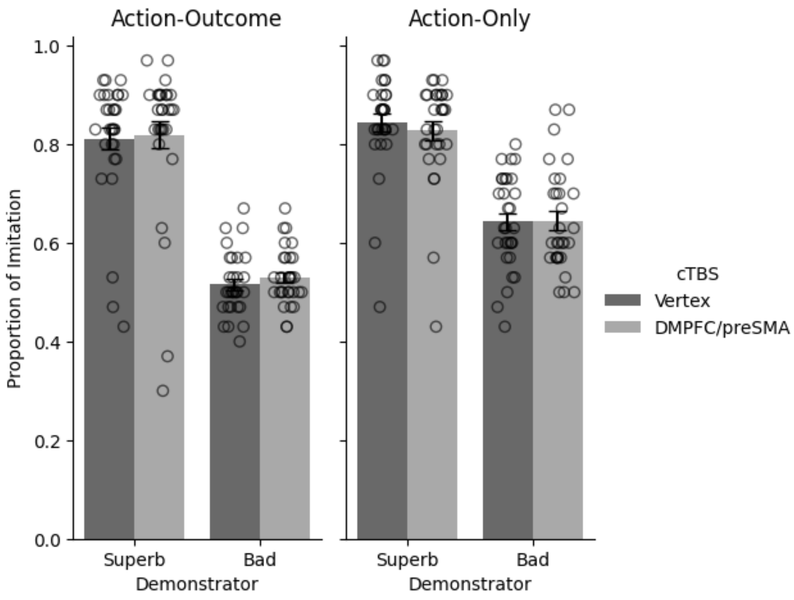

Figure S3. Proportion of imitation behavior. Compared to vertex stimulation, there was little evidence to suggest that DMPFC/preSMA downregulation affected the frequency with which participants chose the same option as the demonstrator on a given trial. Error bars indicate the standard error (SE).

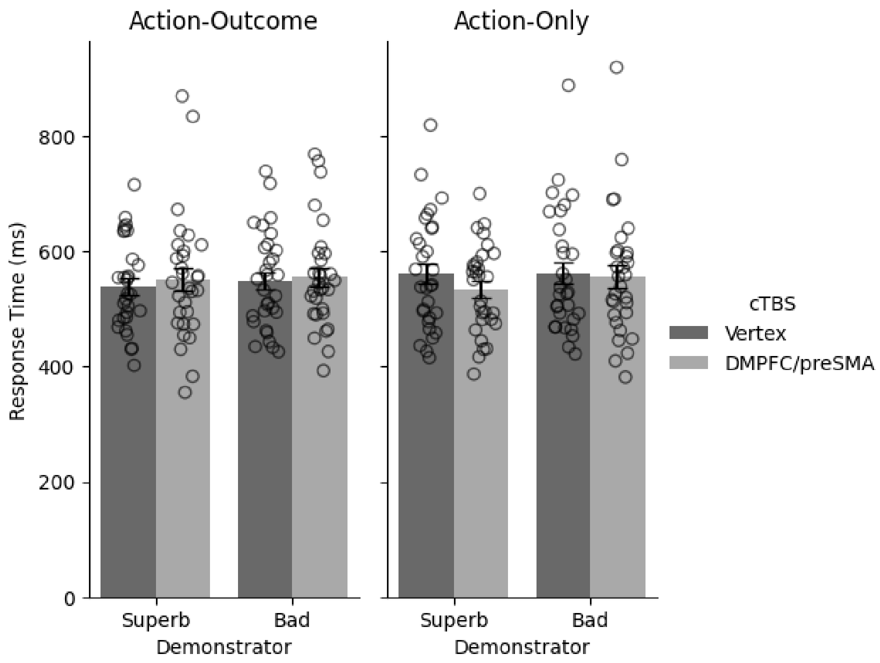

Figure S4. Prediction of demonstrator actions response times. Compared to vertex stimulation, there was little evidence to suggest that DMPFC/preSMA downregulation affected how long it took participants to predict the choice of the demonstrator. Error bars indicate the standard error (SE).

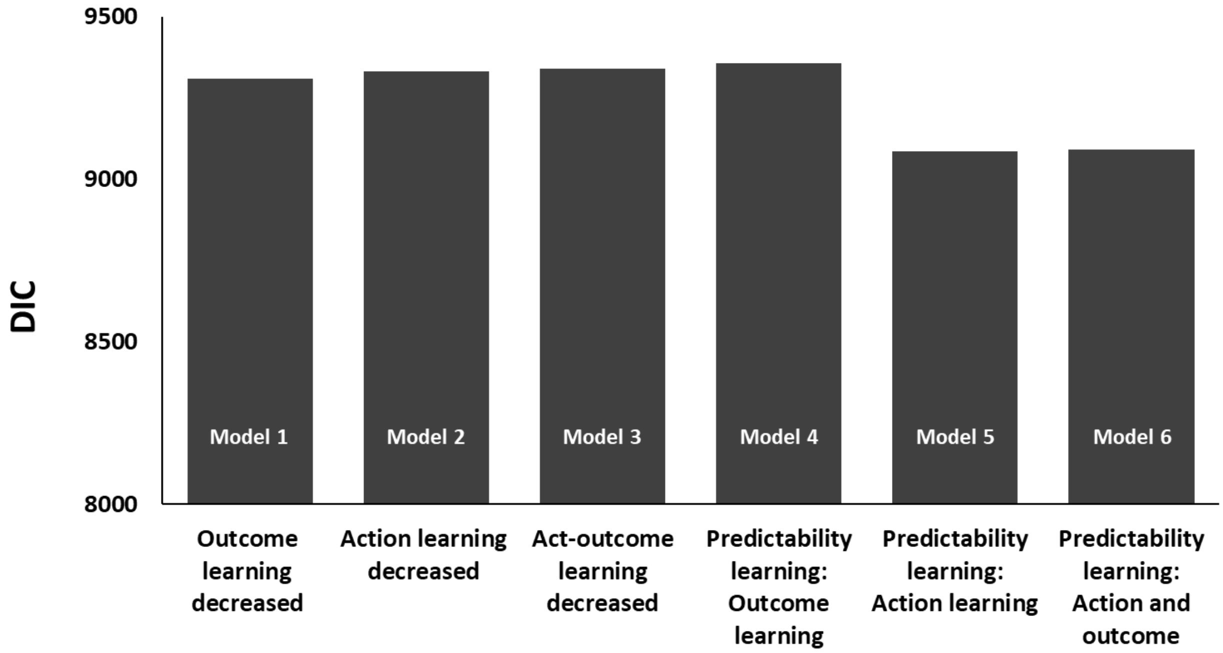

Figure S5. Model comparison for *prediction of demonstrator actions*. Lower DIC values indicate better model fit. The model where DMPFC/preSMA downregulation affected predictability learning in the Action-Only condition explained *prediction of demonstrator actions* best.

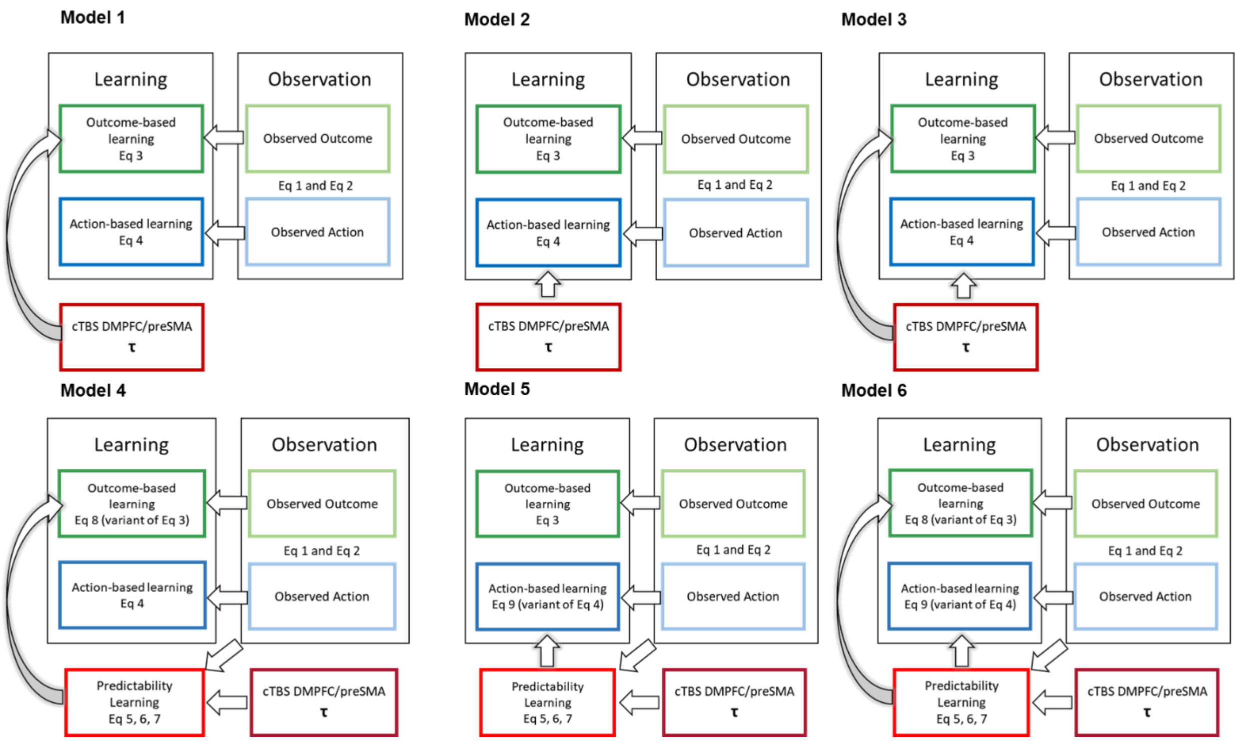

Figure S6. Schematic of the compared models including the equations involved in each model.

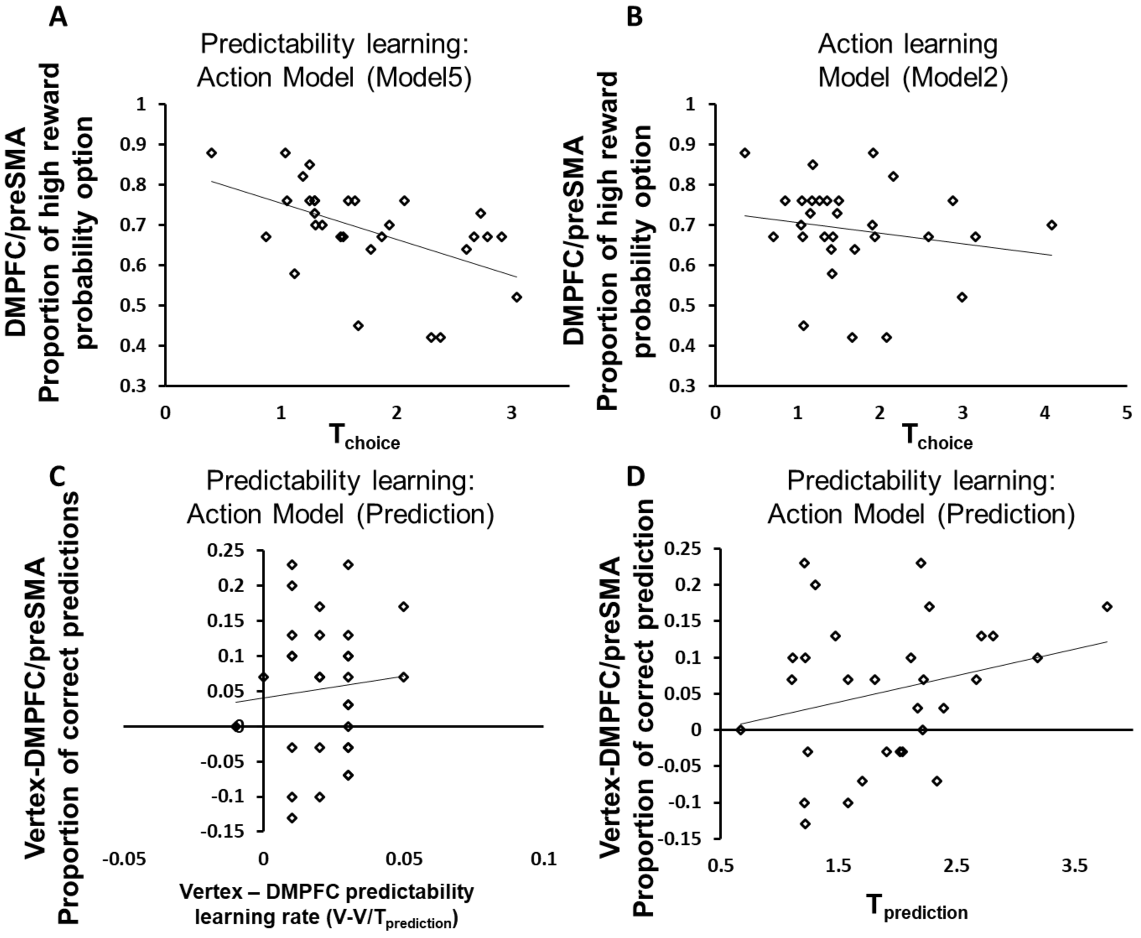

Figure S7. Correlations between model parameters and *choice for self* (A-B) or *prediction of demonstrator actions* (C and D). (A) The stimulation parameter τ_{choice} (from the Predictability action model) correlated significantly with the proportion of choosing the higher reward probability options in the DMPFC/preSMA condition, Pearson's r = -.52, p=0.002 (B) The equivalent analysis revealed no significant correlation for the Action Learning model (Model 2), Pearson's r = -.18, p=0.17. (C) The difference in the proportion of correct predictions between DMPFC/preSMA cTBS and vertex was not significantly related to difference in learning rate between the vertex and DMPFC/preSMA cTBS conditions, Pearson's r = .09, p=0.32. (D) The stimulation parameter $\tau_{prediction}$ related positively to the difference in the proportion of correct predictions between DMPFC/preSMA cTBS and vertex, Pearson's r = .26, p=0.08.

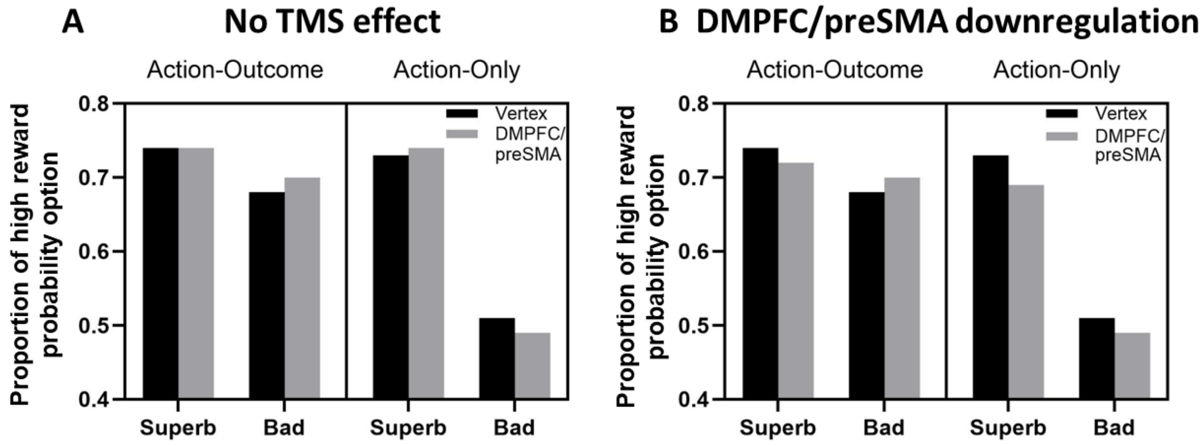

Figure S8. Simulation of DMPFC/preSMA downregulation with the Predictability learning-Action model. A) Simulation of the Predictability learning-Action model with no TMS effect (τ = 1). B) Simulation of the Predictability learning-Action model with DMPFC/preSMA downregulation (τ = 5). With smaller τ, the DMPFC/preSMA condition in the Action-Only condition with the superb demonstrator showed a lower proportion of choosing the higher reward probability option compared to the vertex condition.

Figure S9. Confusion matrix of models for (A) *choice for self* and (B) *prediction of demonstrator actions*

A

Fit model (columns); Simulated model (rows)

	Model 1	Model 2	Model 3	Model 4	Model 5	Model 6
Model 1	1	0	0	0	0	0
Model 2	0.1	0.76	0.14	0	0	0
Model 3	0.08	0.14	0.78	0	0	0
Model 4	0	0.14	0	0.86	0	0
Model 5	0.02	0	0.12	0	0.8	0.06
Model 6	0	0	0	0.16	0.08	0.76

B

Fit model (columns); Simulated model (rows)

	Model 1	Model 2	Model 3	Model 4	Model 5	Model 6
Model 1	0.24	0.18	0.06	0.24	0.22	0.06
Model 2	0.16	0.28	0.02	0.16	0.36	0.02
Model 3	0.22	0.12	0.04	0.22	0.34	0.06
Model 4	0	0	0	1	0	0
Model 5	0.02	0	0	0	0.96	0.02
Model 6	0	0	0	0.04	0.04	0.92

Figure S10. Posterior distributions of the parameters of Model 5. A) Posterior distributions in Model 5 in *choice for self*. B) Posterior distributions of the parameters in Model 5 in prediction of demonstrator actions.

A

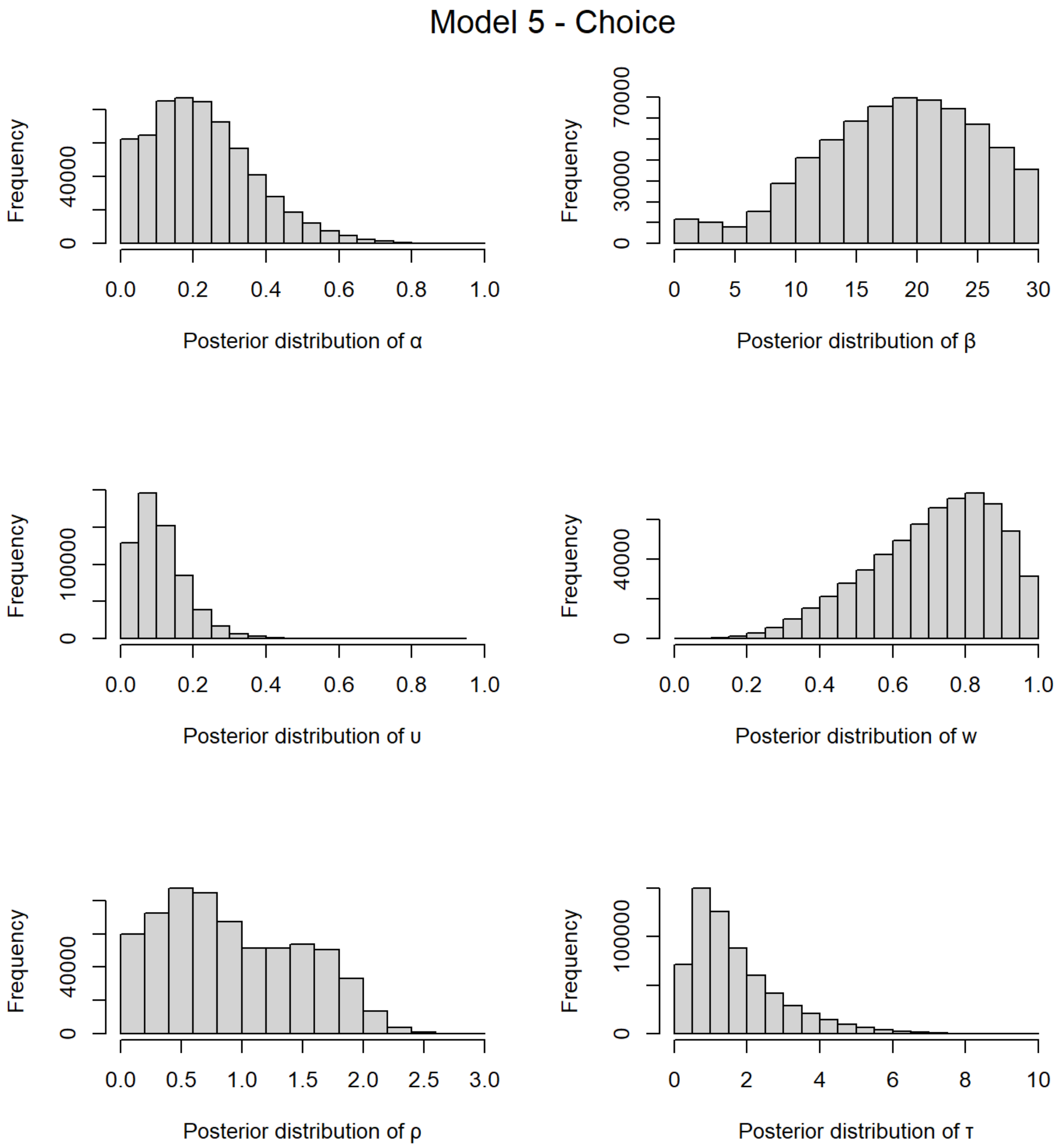

B

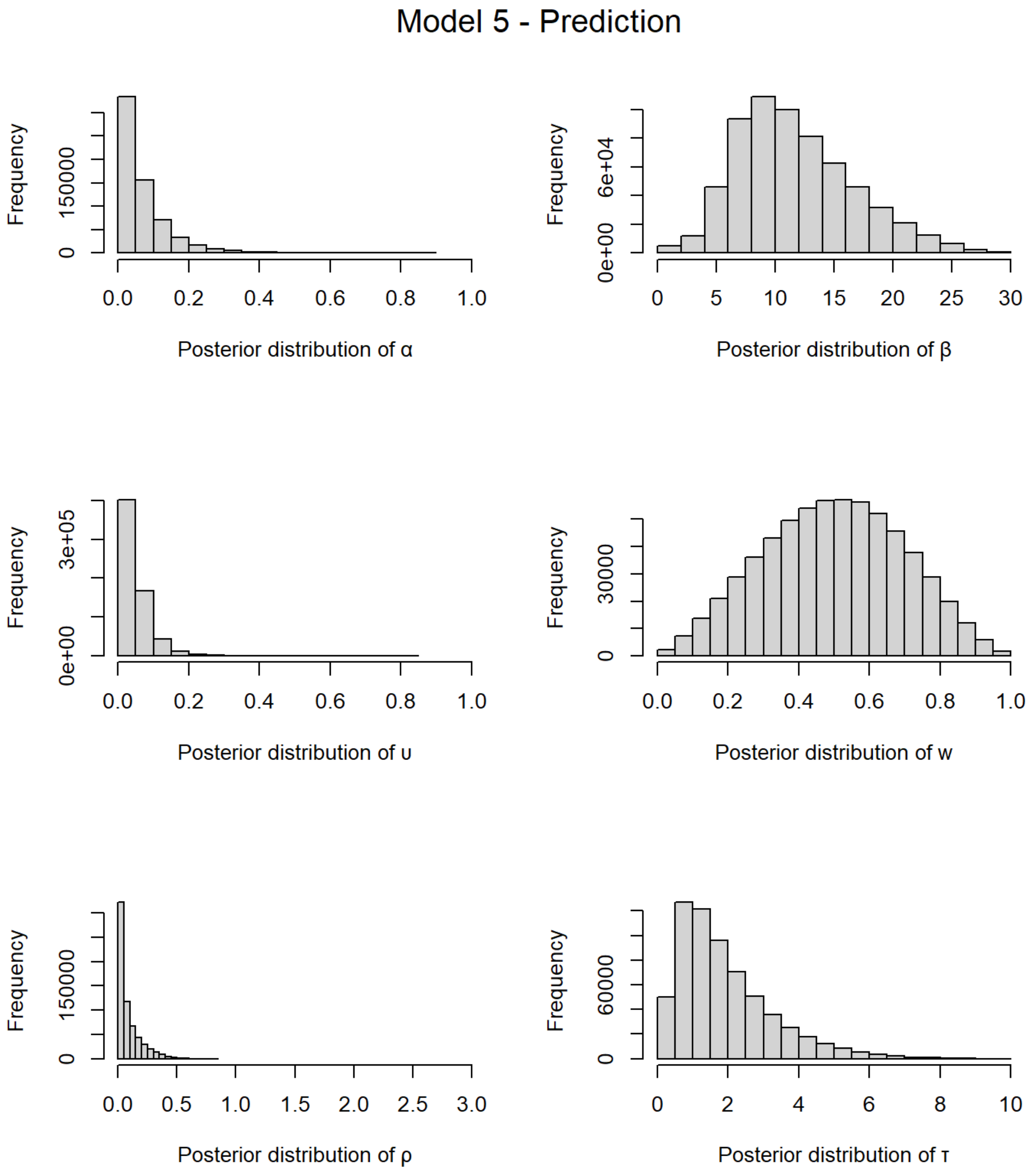
Model 5 - Prediction
Frequency
Posterior distribution of α
Posterior distribution of β
Posterior distribution of υ
Posterior distribution of w
Posterior distribution of ρ
Posterior distribution of τ

Supplementary Tables

Table S1. Parameter recovery. The simulated and recovered parameters of Model5 showed correlations greater than 0.8 for *choice for self* and above 0.6 for *prediction of demonstrator actions*.

	α	β	υ	τ	ρ	ω
Model5 Choice	0.94	0.94	0.95	0.93	0.99	0.84
Model5 Prediction	0.82	0.67	0.64	0.78	0.64	0.84

Table S2. Correlations between the measurements and the learning performance in each condition

	Interpersonal reactivity index	Machiavellianism	Raven test
Action-Outcome Superb Vertex	0.16	0.048	0.053
Action-Outcome Bad Vertex	-0.169	0.202	-0.025
Action-Only Superb Vertex	0.039	0.089	-0.209
Action-Only Bad Vertex	0.106	-0.101	0.142
Individual learning Vertex	0.275	-0.079	0.102
Action-Outcome Superb DMPFC/preSMA	0.044	0.075	-0.14
Action-Outcome Bad	0.126	-0.216	0.172
Action-Only Superb DMPFC/preSMA	0.185	0.306	-0.235
Action-Only Bad DMPFC/preSMA	0.115	-0.043	0.337
Individual learning DMPFC/preSMA	0.218	-0.15	0.01
Action-Outcome Superb Vertex-DMPFC/preSMA	0.065	-0.029	0.143
Action-Outcome Bad Vertex-DMPFC/preSMA	-0.19	0.267	-0.12
Action-Only Superb Vertex-DMPFC/preSMA	-0.127	-0.194	0.057
Action-Only Bad Vertex-DMPFC/preSMA	-0.024	-0.028	-0.173
Individual learning Vertex-DMPFC/preSMA	0.002	0.068	0.057

Table S3. Parameters in each model

	Models	Parameters	Parameters affected by τ (DMPFC/preSMA TMS)
Model 1	Outcome learning decreased	$\alpha,\ \beta,\ \kappa,\ w,\ \rho,\ \tau$	α
Model 2	Action learning decreased	$\alpha,\ \beta, \kappa,\ w,\ \rho,\ \tau$	κ
Model 3	Action-outcome learning decreased	$\alpha,\ \beta,\ \kappa,\ w,\ \rho,\ \tau$	α, κ
Model 4	Predictability learning-Outcome	$\alpha,\ \beta,\ \upsilon,\ w,\ \rho,\ \tau$	υ *(affecting outcome based learning)*
Model 5	Predictability learning - Action	$\kappa,\ \beta,\ \upsilon,\ w,\ \rho,\ \tau$	υ *(affecting action based learning)*
Model 6	Predictability learning - both	$\upsilon, \beta, w,\ \rho,\ \tau$	υ *(affecting action and outcome based learning)*

Table S4. Description of the parameters in computational models

Parameters	Description	
α	Outcome based learning rate	
β	Softmax inverse temperature	
κ	Action-based learning rate	
w	Parameter modulating the weight between outcome- and action-based learning in Action-Outcome trials	
ρ	Perseverance parameter	
υ	Learning rate for predictability of the demonstrators	Only in Model 4,5,6
τ	Parameter for the DMPFC cTBS effect	

Table S5. Model comparisons (DIC) of Models 4-6 to determine optimal alpha and beta of demonstrators

Beta	Alpha	Model 4	Model 5	Model 6
1	0.1	6747.94	6521.257	6999.145
2	0.1	6603.942	6341.256	6671.202
3	0.1	6565.05	6240.218	6509.514
4	0.1	6541.75	6173.442	6412.207
5	0.1	6534.333	6149.626	6369.11
6	0.1	6515.738	6139.623	6366.919
7	0.1	6508.835	6101.817	6345.298
8	0.1	6515.875	6113.723	6345.246
9	0.1	6538.312	6114.926	6340.558
10	0.1	6547.382	6121.371	6358.262
1	0.2	6621.946	6344.984	6649.175
2	0.2	6564.507	6172.838	6432.373
3	0.2	6534.385	6133.271	6399.831
4	0.2	6544.654	6093.83	6389.639
5	0.2	6552.621	6105.8	6387.422
6	0.2	6560.638	6090.231	6392.113
7	0.2	6549.543	6094.046	6411.119
8	0.2	6558.948	6089.398	6404.292
9	0.2	6570.394	**6086.845**	6412.305
10	0.2	6546.346	6087.748	6427.336
1	0.3	6603.193	6247.839	6542.58
2	0.3	6573.331	6132.319	6438.524
3	0.3	6562.381	6109.627	6456.405
4	0.3	6579.105	6109.019	6464.626
5	0.3	6569.286	6098.276	6472.486
6	0.3	6573.051	6091.755	6478.319
7	0.3	6576.158	6099.483	6481.797
8	0.3	6570.271	6099.278	6506.675
9	0.3	6567.553	6094.741	6502.432
10	0.3	6568.03	6100.172	6491.372
1	0.4	6627.748	6220.095	6519.3
2	0.4	6622.202	6130.739	6541.699
3	0.4	6634.926	6128.296	6551.524
4	0.4	6655.349	6133.924	6559.684
5	0.4	6652.81	6121.55	6571.001
6	0.4	6667.795	6114.026	6555.085
7	0.4	6671.233	6140.604	6558.772
8	0.4	6654.218	6126.193	6568.321
9	0.4	6655.274	6120.563	6577.612
10	0.4	6653.001	6118.368	6569.763
1	0.5	6695.706	6239.265	6603.938
2	0.5	6712.873	6178.575	6627.308
3	0.5	6726.444	6162.717	6641.159
4	0.5	6709.941	6164.483	6640.158
5	0.5	6723.522	6144.326	6658.849
6	0.5	6717.57	6165.723	6665.559
7	0.5	6723.334	6160.01	6665.01
8	0.5	6724.677	6152.878	6654.504
9	0.5	6725.564	6145.676	6651.143
10	0.5	6701.796	6160.566	6649.439
1	0.6	6774.566	6253.126	6690.012
2	0.6	6766.972	6221.639	6699.352
3	0.6	6776.676	6194.275	6698.833
4	0.6	6800.024	6200.25	6704.684
5	0.6	6801.586	6193.047	6711.315
6	0.6	6772.027	6198.857	6717.874
7	0.6	6793.324	6208.962	6699.301
8	0.6	6764.746	6192.598	6709.063
9	0.6	6778.156	6209.789	6726.363
10	0.6	6790.819	6193.382	6706.925
1	0.7	6805.597	6308.878	6716.92
2	0.7	6870.746	6268.405	6754.942
3	0.7	6856.434	6258.2	6751.493
4	0.7	6886.547	6244.51	6756.641
5	0.7	6867.582	6242.204	6768.224
6	0.7	6866.767	6251.354	6768.112
7	0.7	6872.517	6237.552	6766.597
8	0.7	6842.732	6250.054	6757.329
9	0.7	6855.253	6235.387	6761.453
10	0.7	6851.159	6248.085	6756.129
1	0.8	6847.836	6367.928	6756.107
2	0.8	6916.439	6321.454	6781.651
3	0.8	6902.682	6306.809	6795.312
4	0.8	6916.867	6302.499	6800.307
5	0.8	6922.286	6291.34	6807.88
6	0.8	6971.94	6303.245	6794.646
7	0.8	6957.419	6273.731	6807.865
8	0.8	6942.138	6298.293	6801.92
9	0.8	6926.607	6296.041	6795.085
10	0.8	6950.409	6301.26	6812.843
1	0.9	6817.974	6417.932	6775.003
2	0.9	6864.049	6388.668	6801.541
3	0.9	6903.975	6375.768	6807.939
4	0.9	6942.761	6374.748	6828.841
5	0.9	6994.533	6359.959	6836.528
6	0.9	6982.07	6348.466	6847.772
7	0.9	6975.73	6351.51	6823.406
8	0.9	7019.292	6357.479	6849.442
9	0.9	7068.835	6349.178	6835.515
10	0.9	7009.447	6346.647	6840.79
1	1	6863.658	6485.376	6795.435
2	1	6855.906	6477.979	6812.688
3	1	6856.123	6479.946	6802.894
4	1	6855.509	6487.636	6804.984
5	1	6855.509	6487.636	6805.219
6	1	6855.509	6487.636	6805.219
7	1	6855.509	6487.636	6805.219
8	1	6855.509	6487.636	6804.984
9	1	6855.509	6487.636	6804.984
10	1	6855.509	6487.636	6804.984

1

Table S6. Model 5: Grid search (DIC) to determine the optimal alpha and beta of demonstrators. Various combinations of alpha, ranging from 0.1 to 1 with increments of 0.1, and beta, from 1 to 10 with increments of 1, were explored to identify the optimal parameter combination for model performance. The findings indicated that the best model fit is achieved with an alpha value of 0.2 and a beta value of 9.

Beta

Alpha

-	1	2	3	4	5	6	7	8	9	10
0.1	6521.26	6341.26	6240.22	6173.44	6149.63	6139.62	6101.82	6113.72	6114.93	6121.37
0.2	6344.98	6172.84	6133.27	6093.83	6105.8	6090.23	6094.05	6089.4	6086.84	6087.75
0.3	6247.84	6132.32	6109.63	6109.02	6098.28	6091.75	6099.48	6099.28	6094.74	6100.17
0.4	6220.1	6130.74	6128.3	6133.92	6121.55	6114.03	6140.6	6126.19	6120.56	6118.37
0.5	6239.26	6178.57	6162.72	6164.48	6144.33	6165.72	6160.01	6152.88	6145.68	6160.57
0.6	6253.13	6221.64	6194.27	6200.25	6193.05	6198.86	6208.96	6192.6	6209.79	6193.38
0.7	6308.88	6268.4	6258.2	6244.51	6242.2	6251.35	6237.55	6250.05	6235.39	6248.08
0.8	6367.93	6321.45	6306.81	6302.5	6291.34	6303.24	6273.73	6298.29	6296.04	6301.26
0.9	6417.93	6388.67	6375.77	6374.75	6359.96	6348.47	6351.51	6357.48	6349.18	6346.65
1.0	6485.38	6477.98	6479.95	6487.64	6487.64	6487.64	6487.64	6487.64	6487.64	6487.64

Supplementary methods

In order to examine whether DMPFC/preSMA cTBS affected imitation behavior instead of action-based learning, we fitted a variation of model 2 to all *choice for self* data in observational conditions. By changing Eq 4 as detailed below, we investigated whether the tendency of choosing the option chosen by a demonstrator is influenced by DMPFC/preSMA cTBS. The model fit was 7349, which is worse than that of models 1-6.

Eq 4. $$AP(a)_i = AP(a)_i + \kappa * (A_i^{demonstrator} - AP(a)_i)$$

Imitation-V $$AP(a)_i = weighting\ of\ imitation * A_i^{demonstrator}$$

Imitation-V $$AP(a)_i = \tau * weighting\ of\ imitation * A_i^{demonstrator}$$

$$A_i^{demonstrator} \begin{cases} option\ chosen\ by\ a\ demonstrator : 1 \\ if\ not : 0 \end{cases}$$
