# Supplementary material for: Causal involvement of dorsomedial prefrontal cortex in learning the predictability of observable actions
Source: Nat Commun. 2024 Sep 27;15:8305. doi: 10.1038/s41467-024-52559-0 (PMC11436984; doi:10.1038/s41467-024-52559-0)
Supplement: Supplementary file 3 — Reporting Summary [file 41467_2024_52559_MOESM3_ESM.pdf]

nature portfolio

Corresponding author(s): Pyungwon Kang & Philippe Tobler

Last updated by author(s): 04/04/2024

# Reporting Summary

Nature Portfolio wishes to improve the reproducibility of the work that we publish. This form provides structure for consistency and transparency in reporting. For further information on Nature Portfolio policies, see our Editorial Policies and the Editorial Policy Checklist.

## Statistics

For all statistical analyses, confirm that the following items are present in the figure legend, table legend, main text, or Methods section.

| n/a | Confirmed | |
|---|---|---|
| ☐ | ☒ | The exact sample size (*n*) for each experimental group/condition, given as a discrete number and unit of measurement |
| ☐ | ☒ | A statement on whether measurements were taken from distinct samples or whether the same sample was measured repeatedly |
| ☐ | ☒ | The statistical test(s) used AND whether they are one- or two-sided<br>*Only common tests should be described solely by name; describe more complex techniques in the Methods section.* |
| ☐ | ☒ | A description of all covariates tested |
| ☐ | ☒ | A description of any assumptions or corrections, such as tests of normality and adjustment for multiple comparisons |
| ☐ | ☒ | A full description of the statistical parameters including central tendency (e.g. means) or other basic estimates (e.g. regression coefficient) AND variation (e.g. standard deviation) or associated estimates of uncertainty (e.g. confidence intervals) |
| ☐ | ☒ | For null hypothesis testing, the test statistic (e.g. *F*, *t*, *r*) with confidence intervals, effect sizes, degrees of freedom and *P* value noted<br>*Give P values as exact values whenever suitable.* |
| ☐ | ☒ | For Bayesian analysis, information on the choice of priors and Markov chain Monte Carlo settings |
| ☒ | ☐ | For hierarchical and complex designs, identification of the appropriate level for tests and full reporting of outcomes |
| ☐ | ☒ | Estimates of effect sizes (e.g. Cohen's *d*, Pearson's *r*), indicating how they were calculated |

*Our web collection on statistics for biologists contains articles on many of the points above.*

## Software and code

Policy information about availability of computer code

| | |
|---|---|
| Data collection | Brainsight frameless stereotaxy (Rogue Research), cogent in Matlab 2018 (Mathworks) |
| Data analysis | “lme4” ,“optimx” "r2jag" and "BayesFactor" packages in R (v3.7.1; www.r-project.org) |

For manuscripts utilizing custom algorithms or software that are central to the research but not yet described in published literature, software must be made available to editors and reviewers. We strongly encourage code deposition in a community repository (e.g. GitHub). See the Nature Portfolio guidelines for submitting code & software for further information.

## Data

Policy information about availability of data

All manuscripts must include a data availability statement. This statement should provide the following information, where applicable:

- Accession codes, unique identifiers, or web links for publicly available datasets
- A description of any restrictions on data availability
- For clinical datasets or third party data, please ensure that the statement adheres to our policy

The data of this study and the code to generate the results is available on https://osf.io/frua8/

## Research involving human participants, their data, or biological material

Policy information about studies with human participants or human data. See also policy information about sex, gender (identity/presentation), and sexual orientation and race, ethnicity and racism.

| | |
|---|---|
| Reporting on sex and gender | We had eighteen female participants and twelve male participants. However, we did not analyze the effect of sex as it was not the focus of our research.<br>Although the participants were WEIRD, considering that brain stimulation is not particularly associated with those participants and that observational learning is a universal phenomenon, it seems unlikely that the results would have been impacted by the sample selection. |
| Reporting on race, ethnicity, or other socially relevant groupings | We have not used any socially relevant groupings in the study. |
| Population characteristics | Thirty-one healthy volunteers (18 females, age average = 23.59±2.45) were recruited from the University of Zurich and the Swiss Federal Institute of Technology in Zurich. |
| Recruitment | Participants were recruited through an online recruitment system in the department where the researchers belong to. |
| Ethics oversight | the Research Ethics Committee of the canton of Zurich. |

Note that full information on the approval of the study protocol must also be provided in the manuscript.

# Field-specific reporting

Please select the one below that is the best fit for your research. If you are not sure, read the appropriate sections before making your selection.

☐ Life sciences ☒ Behavioural & social sciences ☐ Ecological, evolutionary & environmental sciences

For a reference copy of the document with all sections, see nature.com/documents/nr-reporting-summary-flat.pdf

# Behavioural & social sciences study design

All studies must disclose on these points even when the disclosure is negative.

| | |
|---|---|
| Study description | quantitative experimental design |
| Research sample | Thirty-one healthy volunteers (18 females, age average = 23.59±2.45) were recruited from the University of Zurich and the Swiss Federal Institute of Technology in Zurich. Eligibility for TMS was determined based on standardized safety guidelines (Rossi et al., 2009). |
| Sampling strategy | Power analyses indicated that a minimum of thirty participants was needed to obtain a statistical power of 80% for detecting significant TMS effects (assuming a standard significance criterion of $\alpha$ = 5%) in a within-subject design, with the same moderate effect size (d=0.67) of previous TMS research (Soutschek et al., 2016). |
| Data collection | The behavioral data was collected with a computer while participants performed a learning task after brain stimulation in a session. It was a within-subject design, the same procedure repeated twice. The experimenter was aware of the condition as the stimulation was manually given by the experimenter. |
| Timing | The data collection started on 19th October 2018 and finished 19 February 2019. |
| Data exclusions | No data were excluded. |
| Non-participation | *State how many participants dropped out/declined participation and the reason(s) given OR provide response rate OR state that no participants dropped out/declined participation.* |
| Randomization | The experiment was a within subject design, thus, there was no allocations to groups. The order of stimulation was randomized. |

# Reporting for specific materials, systems and methods

We require information from authors about some types of materials, experimental systems and methods used in many studies. Here, indicate whether each material, system or method listed is relevant to your study. If you are not sure if a list item applies to your research, read the appropriate section before selecting a response.

## Materials & experimental systems

| n/a | Involved in the study |
| --- | --- |
| ☒ | ☐ Antibodies |
| ☒ | ☐ Eukaryotic cell lines |
| ☒ | ☐ Palaeontology and archaeology |
| ☒ | ☐ Animals and other organisms |
| ☒ | ☐ Clinical data |
| ☒ | ☐ Dual use research of concern |
| ☒ | ☐ Plants |

## Methods

| n/a | Involved in the study |
| --- | --- |
| ☒ | ☐ ChIP-seq |
| ☒ | ☐ Flow cytometry |
| ☒ | ☐ MRI-based neuroimaging |
